# Gene expression profile of human lung epithelial cells chronically exposed to single-walled carbon nanotubes

**DOI:** 10.1186/s11671-014-0707-0

**Published:** 2015-01-27

**Authors:** Dongquan Chen, Todd A Stueckle, Sudjit Luanpitpong, Yon Rojanasakul, Yongju Lu, Liying Wang

**Affiliations:** Division of Preventive Medicine, Department of Medicine, University of Alabama at Birmingham, Birmingham, AL 35294 USA; Pathology and Physiology Research Branch, National Institute for Occupational Safety and Health, Morgantown, WV 26505 USA; Department of Pharmaceutical Sciences and Mary Babb Randolph Cancer Center, West Virginia University, Morgantown, WV 26506 USA; Department of Pharmaceutical Sciences, Wayne State University, Detroit, MI 48201 USA

**Keywords:** Single-walled carbon nanotubes, Lung, Microarray, Gene expression, Pathways, p53

## Abstract

A rapid increase in utility of engineered nanomaterials, including carbon nanotubes (CNTs), has raised a concern over their safety. Based on recent evidence from animal studies, pulmonary exposure of CNTs may lead to nanoparticle accumulation in the deep lung without effective clearance which could interact with local lung cells for a long period of time. Physicochemical similarities of CNTs to asbestos fibers may contribute to their asbestos-like carcinogenic potential after long-term exposure, which has not been well addressed. More studies are needed to identify and predict the carcinogenic potential and mechanisms for promoting their safe use. Our previous study reported a long-term *in vitro* exposure model for CNT carcinogenicity and showed that 6-month sub-chronic exposure of single-walled carbon nanotubes (SWCNT) causes malignant transformation of human lung epithelial cells. In addition, the transformed cells induced tumor formation in mice and exhibited an apoptosis resistant phenotype, a key characteristic of cancer cells. Although the potential role of p53 in the transformation process was identified, the underlying mechanisms of oncogenesis remain largely undefined. Here, we further examined the gene expression profile by using genome microarrays to profile molecular mechanisms of SWCNT oncogenesis. Based on differentially expressed genes, possible mechanisms of SWCNT-associated apoptosis resistance and oncogenesis were identified, which included activation of pAkt/p53/Bcl-2 signaling axis, increased gene expression of Ras family for cell cycle control, Dsh-mediated Notch 1, and downregulation of apoptotic genes *BAX* and Noxa. Activated immune responses were among the major changes of biological function. Our findings shed light on potential molecular mechanisms and signaling pathways involved in SWCNT oncogenic potential.

**Electronic supplementary material:**

The online version of this article (doi:10.1186/s11671-014-0707-0) contains supplementary material, which is available to authorized users.

## Background

Carbon nanotubes (CNT) are among the most widely used nanomaterials for various industrial and biomedical applications [1-3]. Their unique properties of high tensile strength, flexibility, thermal and electrical conductivity, and their light weight have contributed to their widespread use. However, their rapid growth in utility has raised a major concern about their safety, especially on human health and environment. CNT share several properties with asbestos fibers such as high aspect ratio, durability, biopersistence, and mode of exposure (e.g., inhalation). Because asbestos fibers are classified as a Group I human carcinogen by the International Agency of Research in Cancer [4], there is a concern about the potential carcinogenicity of CNT [2]. Exposure to asbestos has been shown to cause various lung diseases including lung cancer and mesothelioma [5]. Thus, there is a clear need for carcinogenicity studies of CNT to develop control and prevention strategies [6].

Recent studies have shown that both single-walled (SW) and multi-walled (MW) CNT deposit in the deep lung tissue of mice with low clearance after pulmonary administration [7-9]. Occupationally relevant *in vivo* exposures (10 to 80 μg/mouse) and pulmonary fibroblast *in vitro* exposure studies, using *in vivo* dose equivalents (0.02 to 0.2 μg/cm^2^), resulted in dose-dependent transient pulmonary inflammation followed by fibroblast cell proliferation, alveolar wall thickening, and collagen I production culminating in persistent pulmonary fibrosis [9,10]. Several *in vitro* and *in vivo* studies have suggested the potential carcinogenicity of CNT [11-13]. The possible carcinogenic mechanisms include DNA damage [14-16], mitotic disruption [12], impaired apoptosis, and activation of oncogenic signaling events, which recapitulate asbestos-induced lung cancer and mesothelioma [17-19]. It was suggested that nanotube bundles are similar in size to microtubules that form the mitotic spindle and may be incorporated into the mitotic spindle apparatus, resulting in multipolar mitosis and aneuploidy [20] which were observed in asbestos-treated cells [15]. Furthermore, CNT exposure induced asbestos-like granulomas in mice in a short-term abdominal instillation study [18].

Multiple genes may be involved in single-walled carbon nanotubes (SWCNT) pathogenesis. For examples, p38 MAPK was reported to regulate SWCNT-induced fibrogenic and angiogenic responses [21]. Other short-term exposure studies report increased NF-kβ, TNFα, NRLP3, iNOS, p53, and TGFβ activity all culminating in lung inflammation and the onset of pulmonary interstitial fibrosis [22,23]. Long-term *in vitro* and *in vivo* exposure studies found that SWCNT and MWCNT exposure to lung tissues resulted in *KRAS* activation, enhanced micronuclei and nuclear protrusions, mitosis disruption, mutated p16, cMyc proto-oncogene signaling, and enhanced metalloproteinase remodeling of the extracellular matrix [9,24-27], which all indicate tumorigenesis potential following CNT exposure. Recent transcriptomic and sequencing studies suggest that long-term CNT exposure results in similar signaling compared to other biopersistent fibers (i.e., asbestos) but also display unique signaling pathways [27-30] raising the possibility of a distinct CNT pathogenesis paradigm [31]. Although a majority of these pro-inflammation and pro-fibrotic genes have known roles in cancer, a consensus on those key molecules and signaling pathways that potentially drive and contribute to CNT-associated carcinogenesis remain elusive.

Our recent studies showed that low-dose (0.02 μg/cm^2^) SWCNT exposure caused malignant transformation and tumorigenesis of human lung bronchial epithelial cells [13]. We also found that phosphorylation at various positions on p53 in these malignant cells significantly decreased after sub-chronic SWCNT exposure [13]. These phosphorylation sites are crucial for stabilization and activation of p53-dependent functions of tumor suppression, DNA damage repair, and apoptosis resistance [32-35]. At present, the upstream and downstream signal transduction pathways associated with altered p53 status contributing to a SWCNT-induced epithelial tumorigenic phenotype are unknown.

Since carcinogenesis is a long-term, multi-step process and involves multiple genes, it is of critical importance to study the chronic exposure and genome-wide expression changes to define the underlying molecular mechanisms of carcinogenesis, which is largely unknown. To accomplish this goal, we have developed a sub-chronic exposure model in which human lung bronchial epithelial cells, the primary target of CNT inhalation exposure, were continuously exposed to low-dose SWCNT in culture over a long period (6 months) [13]. Because isolated primary lung cells do not survive in long-term culture, we employed an immortalized human lung epithelial BEAS-2B cell line which is non-tumorigenic and exhibits similar cellular responses [7,20,36]. The long-term SWCNT-exposed BEAS-2B (B-SWCNT) cells were evaluated, for the first time, for genome-wide expression profiling and functional analysis compared to unexposed, passage-matched control cells. Our study revealed multiple genes and signaling networks that are affected by SWCNT-induced oncogenic transformation which might serve as potential SWCNT-specific exposure or disease markers. This *in vitro* approach also supports prudent adoption of exposure control strategies protection of workers, consumers, and the environment.

## Methods

### Preparation of single-walled carbon nanotubes

SWCNT (CNI, Houston, TX, USA) were produced by the high-pressure CO disproportionation (HiPco) technique, employing CO in a continuous-flow gas phase as the carbon feedstock and Fe(CO)_5_ as the iron-containing catalyst precursor and used in our previous study [13]. Briefly, the SWCNT were purified by acid treatment to remove metal contaminates. Elemental analysis of the supplied SWCNT showed that the SWCNT were 99% elemental carbon and 0.23% iron. The specific surface area measured at −196°C by the nitrogen absorption-desorption technique (Brunauer-Emmet-Teller method) was 400 to 1,000 m^2^/g. The diameter and length distribution of the SWCNT measured by field emission scanning electron microscopy which were 0.8 to 1.2 nm and 0.1 to 1 μm, respectively. SWCNT were dispersed by acetone/sonication method as previously described [10]. Briefly, SWCNT were treated with acetone and placed in an ultrasonic bath for 24 h. The dispersed CNT were then filtered from the solution using a 20-μm nylon mesh screen followed by a 0.2-μm polytetrafluoroethylene filter. After filter collection, the dispersed CNT were washed thoroughly with distilled water and suspended in phosphate-buffered saline (PBS) with 2- to 3-min sonication (Sonic Vibra Cell Sonicator, Sonic & Material Inc, Newtown, CT, USA).

### *In vitro* treatment and cell sample preparation for microarrays

Microarray sample collection and analyses were performed on unexposed and SWCNT-exposed cells from our previous study [13]. Briefly, human lung bronchial epithelial BEAS-2B cells were continuously exposed for 6 months to a sub-cytotoxic concentration (0.02 μg/cm^2^; equivalent to 0.1 μg/ml) of dispersed SWCNT or PBS (non-treatment control) in a six-well culture plate in triplicate. Each replicate was exposed and independently assayed throughout the study. This relatively low concentration was chosen due to its relevance to *in vivo* SWCNT exposure dose of 10 μg/mouse previously reported [8,37,38]. Repeated, long-term dosing of particulate in *in vitro* systems may result in accumulation of particle burden over time in the assay system. To reduce and safeguard against potential SWCNT accumulation over time, every 3 to 4 days, the exposure media was aspirated and the cells were triplicate washed with PBS to remove suspended and unbound SWCNT. Cells were then resupplied with new exposure media containing SWCNT. The cells were passaged weekly at pre-confluent densities using a solution containing 0.05% *w*/*v* trypsin and 0.5 mM EDTA (Invitrogen, Carlsbad, CA, USA). Greater than 50% of cells were removed during passaging which resulted in a relatively small proportion of intracellular SWCNT remaining in the system. Parallel cell cultures grown in the same media without SWCNT provided passage-matched control. Following the 6-month exposure, SWCNT-treated (B-SWCNT) and control (B-Control) cells were evaluated for genome-wide gene expression. Three B-SWCNT cell biological replicates showing similar phenotypic behavior and p53 expression patterns [13] were pooled and analyzed as one. Additional filters such as increasing the fold change to >2 will limit this disadvantage. Since the objective of this study was to explore the candidate genes involved in the carcinogenic process for further studies, this study is considered a preliminary step.

RNA sample preparation and hybridization were performed as previously described [39]. Briefly, total RNA was harvested and isolated using TRIzol (Invitrogen) according to manufacturer's instructions. Isolated RNA in DEPC-treated water was then immediately frozen at −80°C for 24 h and shipped on dry ice to ArrayStar (Rockville, MD, USA) for mRNA processing, microarray hybridization, and probe expression normalization.

### DNA microarray and data analysis

Whole genome oligo microarrays (#014850, Agilent Technology, Santa Clara, CA, USA) were used. Each array represents more than 41,000 unique human genes and transcripts sourced from RefSeq, Goldenpath Ensembl Unigene Human Genome (Build 33) and GenBank databases. Fluorescent RNA targets were prepared using Agilent Quick Amp Labeling Kit. Microarray hybridization was performed at 65°C for 17 h in Agilent's SureHyb hybridization chambers. After washing in an ozone-free environment, the slides were scanned using the Agilent DNA microarray scanner (model G2505B).

Raw data were extracted using Agilent Feature Extraction Software (Santa Clara, CA, USA). Expression raw data from passage control cells were used for other exposure studies. The resulting text files were imported into the Partek Genomic Suite (PGS; St. Louis, MO, USA) for preprocessing, normalization, and statistical analysis. Briefly, normalization was performed using the Agilent FE one-color scenario (mainly median normalization). Genes marked present or marginal in all samples (‘All Targets Value’) were chosen for further data analysis. Differentially expressed genes (DEGs) were identified through fold-change screening and *t*-test assuming unequal variances. Genes were differentially expressed in B-SWCNT cells if they exhibited ≥ ±twofold expression and *p* ≤ 0.05 compared to B-Control cells. Unsupervised hierarchical clustering, gene ontology (GO) analysis, and pathway analysis were performed using the PGS (St. Louis, MO). False discovery rate (FDR) assessment by Benjimini Hochberg methods was applied for multiple hypothesis testing purpose [40]. The filtered gene lists were used for GO, pathways, and gene-gene interaction analyses by using Ingenuity Pathway Analysis (IPA) software package (Redwood City, CA, USA).

### Apoptosis protein array

To substantiate the genomic data and further identify proteins contributing to the apoptotic resistance phenotype, B-SWCNT and B-Control cell protein lysates were subjected to a human apoptosis protein expression array (see Additional file 1: Table S1 for array layout) in duplicate following manufacturer's instructions (R & D Systems, Minneapolis, MN, USA). Briefly, 2 × 10^6^ cells from each treatment were seeded into a 25-cm^2^ flask in particle-free growth medium and cultured for 24 h. Next, plated cells were placed on ice, washed in ice cold PBS, and lysed using the manufacturer's lysis buffer. Cell lysate was collected via rubber policemen, rocked on ice in suspension of 30 min, and spun at 12.5 K × *g* to collect supernatant. Protein levels were determined via BCA absorbance method on a spectrophotometer at 562 nm. Five hundred micrograms of protein was incubated with each protein array overnight at 4°C. Each array was triplicate washed in buffer, incubated with detection antibody for 1 h, washed again, and incubated with streptavidin-HRP antibody for 30 min. Protein expression was captured using HRP chemiluminescence on X-ray film.

### Western blot

Bcl-2, pAkt, and Akt protein expression was determined using previously described Western blot methods [10]. Briefly, B-SWCNT and B-Control cells were seeded (5 × 10^5^ cells/well) and cultured in six-well plates for 24 h in particle-free medium or re-exposed to dispersed SWCNT (1 to 50 μg/ml or 0.1 to 5.2 μg/cm^2^). Cells were placed on ice, washed with ice cold PBS buffer, and lysed in protein buffer containing PMSF and protease inhibitor (Invitrogen). Cells were scraped, collected, and rocked on ice for 30 min. Following centrifugation at 12.5 K × *g*, BCA assay was used to measure protein concentration in collected supernatants. Equal amounts of protein were loaded into SDS PAGE gel, allowed to separate under current, and transferred to PVDF membranes using semi-dry transfer apparatus (Fisher Scientific, Pittsburgh, PA, USA). Membranes were incubated with antibodies to either Bcl-2, pAkt, Akt (Cell Signaling, Beverly, MA, USA), or B-actin (Sigma, St. Louis, MO, USA) overnight at 4°C, washed thrice in Tris buffer with 0.1% Tween, and incubated with anti-rabbit or anti-mouse HRP antibody for 1 h (Santa Cruz, Inc, Paso Robles, CA, USA). Protein levels were determined using Millipore Immobilon HRP chemiluminescent substrate (Millipore, Billerica, MA, USA) and exposure to X-ray film. Western blot experiments were independently performed three times. Quantification of Western blot results were performed by using ImageJ [41,42]. Statistical analyses were performed by using two group Student *t*-tests after quantification and normalization to corresponding controls.

## Results and discussion

As we reported previously [13], transformed B-SWCNT cells displayed aberrant p53 signaling, increased aggressiveness, apoptosis resistance, anchorage-independent growth, angiogenicity, and *in vivo* xenograft tumor formation compared to unexposed passage control cells. These findings suggested the potential carcinogenicity of B-SWCNT cells. The present transcriptomic analysis and supporting data also indicate aberrant p53 signaling, pAkt, Ras, Notch 1, and altered intrinsic mitochondrial pathway signaling as potential mechanisms for SWCNT carcinogenicity. This is consistent with the previous observations that most human cancers possess p53 tumor suppressor gene inactivation resulting in dysfunctional signaling contributing to tumorigenesis [43,44].

### Gene expression profile by hierarchical clustering

**Figure 1:**
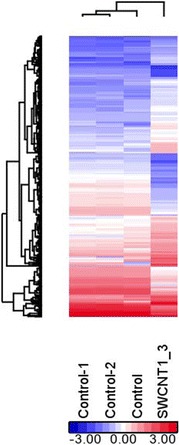
**Hierarchical clustering of SWCNT-exposed and passage control cell gene expression following 6 months of continuous treatment.** The hierarchical clustering was based on differentially expressed genes of SWCNT vs. control comparison using *p* < 0.05 and fold changes >2 criteria. Color indicates log2-transformed normalized intensities with red and blue indicating over- and underexpression, respectively.

Hierarchical clustering is a convenient way to visualize the overall similarity among a large pool of samples. The heatmap pattern also offers clues of possible co-expression of genes. As shown in Figure 1, three control samples showed similar gene expression patterns to cluster on the left (control, control-1, and control-2). The B-SWCNT cells possessed a different expression pattern on the right (SWCNT1_3). Here, three treated samples were pooled and analyzed as one. This is a cost-effective way to get the most likely candidate genes but with the disadvantage of lowering the power and introducing more false positives. Since the objective of this study was to explore the candidate genes involved in the carcinogenic process for further studies, this study is considered a preliminary step.

### Gene ontology (GO) analysis for biological processes and molecular functions

We analyzed top GO biological processes that are affected by SWCNT exposure based on the list of DEGs in B-SWCNT cells. Genes that are involved in immune response were among the top biological processes (Table 1). The top biological processes include the responses to metal ion, antigen process and presenting, regulation of phagocytosis, and vacuolar transport. Apparently, lung epithelial cells may recognize SWCNT as foreign which can alter the innate immune response and influence the immune system as reported [27,45,46]. In addition, the positive regulation of microtubule depolymerization suggests that long-term SWCNT exposure altered microtubule dynamics as previously reported [12,47]. Similarly, genome wide changes of GO molecular functions after SWCNT exposure included genes responsible for immune response (i.e., MHC class I) protein binding, post-transcriptional modifications (e.g., ubiquitin protease activity), and other enzymatic activities were among the top molecular function changes (Table 2). Previous ‘omics’ studies using long-term SWCNT exposure *in vivo* or *in vitro* models reported similar findings that protein post-translational modification constitutes a large GO signal [27,28] and is likely to play a large role in disease development and pathology.

**Table 1:** **Top biological processes changed after SWCNT treatment in transformed BEAS-2B cells compared to unexposed cells**

**Biological process**	**Enrichment score**	**Enrichment** ***p*** **value**	**Percent of genes in group that are present**	**GO ID**
Response to metal ion	8.6	0.000	37.5	10038
Antigen processing and presentation of peptide antigen via MHC class I	8.5	0.000	8.6	2474
Regulation of circadian rhythm	7.9	0.000	30	42752
Antigen processing and presentation	7.4	0.001	10.9	19882
Positive regulation of microtubule depolymerization	7.3	0.001	66.7	31117
Regulation of phagocytosis	7.3	0.001	66.7	50764
Interspecies interaction between organisms	7	0.001	4.2	44419
Cellular chloride ion homeostasis	6.6	0.001	50	30644
Vacuolar transport	6.6	0.001	50	7034
Rhythmic process	6.4	0.002	18.8	48511

The biological process was based on SWCNT vs. control comparison, *p* < 0.05 and fold changes >2.

**Table 2:** **Top molecular functions changed after SWCNT treatment in transformed BEAS-2B cells**

**Molecular function**	**Enrichment score**	**Enrichment** ***p*** **value**	**Percent of genes in group that are present**	**GO ID**
MHC class I receptor activity	13.6	0.000	35.7	32393
Flavonol 3-sulfotransferase activity	7.3	0.001	66.7	47894
Aryl sulfotransferase activity	6.1	0.002	40.0	4062
G-protein coupled photoreceptor activity	6.1	0.002	40.0	8020
SH3 domain binding	6.1	0.002	5.8	17124
Ubiquitin-specific protease activity	5.7	0.003	9.8	4843
Photoreceptor activity	4.9	0.008	22.2	9881
Protein binding	4.7	0.009	1.9	5515
Ubiquitin thiolesterase activity	4.7	0.009	5.8	4221
Cysteine-type endopeptidase activity	4.5	0.011	6.9	4197

The molecular function was based on SWCNT vs. control comparison, *p* < 0.05 and fold changes >2.

### Functional analysis of gene-gene interactions

Multiple gene-gene interactions are important for gene functions and signal transduction. Therefore, gene-gene interaction networks were plotted, scored, and ranked to identify interactions potentially driving the observed B-SWCNT malignant phenotype. Consistent with the observed carcinogenic and apoptosis-resistant phenotype of B-SWCNT cells [13], immune response, cell growth, cell death/survival, and cell cycle control signaling network were among the major functions identified in the top-ranked networks in the B-SWCNT toxicogenomic profile (Table 3). The functional involvement of individual genes or pathways in the carcinogenic process needs to be further studied individually to determine their actual involvement. Regardless, the pathways of *Cellular growth and proliferation, hematological system development and function, humoral immune response*, *cell death and survival, embryonic development, organismal development,* and *cancer*-related gene-gene interaction networks possessed gene-gene interactions known to participate in tumorigenic and CNT-induced neoplastic signaling mechanisms [27].

**Table 3:** **Top affected gene-gene interaction networks after sub-chronic SWCNT exposure in transformed BEAS-2B cells**

**Top diseases and functions**	**Molecules in network** ^**a,b**^	**Score**	**Focus molecules** ^**c**^
Cellular growth and proliferation, hematological system development and function, humoral immune response	ADAM15, ADRBK2, ATP5B, BCL10, BCR (complex), CHGA, CR2, CRHR1, ERK1/2, FGR, GNAZ, HOPX, HSD11B2, HSPD1, HTATIP2, IgG1, Igg3, IgG2b, IGLL1/IGLL5, Igm, insulin, ITCH, Jnk, MFGE8, NEU1, PAK2, PAX4, PPP1R1B, Ras, RASGRP1, SH2B2, SH2D2A, TAS1R1, Vegf, ZNF24	35	25
Connective tissue disorders, inflammatory disease, skeletal and muscular disorders	C1R, CD33, CD59, CDON, G6PD, HLA-A, HLA-B, HLA-C, HLA-E, Ifnar, IgG, IKK (complex), immunoglobulin, interferon alpha, KIR, KRT31, MYO1E, NFAT5, NFkB (complex), P38 MAPK, PI3K (family), PIM1, Pkc(s), RHOB, RNF34, RTKN, SCARB1, SIRPA, SLC6A6, Tap, TAPBP, TNFSF10, TRAF3IP2, USP11, USP18	33	24
Cell death and survival, embryonic development, organismal development	ADAM17, AFF4, AKT2, Akt, AMPK, Ap1, BHLHE40, CD68, CELF1, CSNK1E, CSNK1G2, Cyclin A, DVL1, estrogen receptor, F actin, FMR1, GSK3B, HMGB3, HMGN5, Hsp70, IGFBP2, LCP1, LDL, MGLL, MUC4, OSGIN1, PDGF BB, PI3K (complex), PMAIP1, Ppp2c, PURA, SF3B3, STRN, USP6, USP33	33	24
Cellular compromise, skeletal and muscular system development and function, post-translational modification	ARHGEF9, ATP13A2, CD3, Cg, Creb, CTSB, DUSP9, ERK, FBN1, FGF3, FSHR, GSTM4, Hdac, Histone h3, Histone h4, HSD17B1, IFI44, IFI44L, IgG2a, KDM3A, Lh, MCOLN1, MT1F, MT1X, NDRG1, NDRG4, NES, NPNT, ORAI1, OXT, PGK1, SOX12, STAT5a/b, STK17A, TCR	33	24
Cell morphology, cellular assembly and organization, cellular development	ACBD7, ADAM22, ANK1, AR, AS3MT, ATP5S, ATP6V0E2, ATP8B1, CACNA1E, CDC6, CHD8, DLG4, FARP1, GLUL, GPR182, GSTM1, HILPDA, HIST1H1A, IGF1R, KCNA4, KCNAB2, MCM8, NIPSNAP1, NUPR1, PPT2, RAB18, RCN2, SIPA1L1, SOX11, ST6GALNAC6, SYNGAP1, TEX2, TMEM30A, WNK1, ZSCAN16	21	18
Respiratory system development and function, tissue morphology, cell cycle	26 s Proteasome, ACTC1, ACTN1, ACTR2, ACTR3, BNIP3L, CAP2, CARD10, CKAP2, EBAG9, ELMOD3, ENC1, FAM89B, FSH, GEM, IGLL1/IGLL5, ING2, JMJD6, MED26, MT1L, NOL3, NR3C1, PRPH, PSMB1, RAB11A, RDM1, RNA polymerase II, SCAF8, SERTAD2, SFTPC, SIAH2, SMARCC1, TFDP2, TUBA4A, USP13	16	15
Cancer, gastrointestinal disease, hepatic system disease	ACOT11, AHCY, ANXA7, ARL6IP1, ARNT, ASS1, AXIN1, AXIN2, BCLAF1, CAMLG, CCDC80, CYP1A1, EPHX1, FAM120A, FBXW7, HIST1H1C, IGFBP2, KAT5, KITLG, KREMEN2, MED13L, MT1H, N-cor, NCOR2, NQO1, PIAS2, PSMF1, PTGDS, PTP4A3, RBBP4, SERPINB6, SIDT2, STOX1, TCF7L2, TP53	16	15
Cell morphology, cell-to-cell signaling and interaction, nervous system development and function	AIG1, ALDH3B1, C11orf30, CABYR, CACNA2D1, CHMP4B, COX10, CYFIP2, DGKZ, DMD, DNAJB6, DTNB, DYRK1A, HDAC4, HYOU1, INS, LEPR, Map4k4, MARK3, OPN4, Pdx1, PFKM, PPFIA1, proinsulin, SNCA, SNTB2, SPARC, SPOP, TNRC6B, TRIM44, TUBB4A, VEGFA, VGF, YWHAG, YWHAH	16	15
DNA replication, recombination, and repair, cellular compromise, cell death and survival	ATP6V0E1, BAG1, BAX, BGN, BRCA1, BRCA2, CCND1, CEL, CKB, DDX5, DNAJA1, DPYSL3, FANCD2, GATA1, GFI1B, GSK3A, H3F3A/H3F3B, Hbb-b2, HSPA4, HSPD1, LATS1, LINC00467, MAPT, NGFR, NME4, OPA1, ROCK2, SFN, SPOCK2, STIP1, TLE1, UBE2N, USP11, ZFPM1, ZNF324	15	14
Lipid metabolism, molecular transport, small molecule biochemistry	AP1M2, APOA1, APOC1, ARCN1, CBS, CCL1, CCL22, CETP, CLIC4, COG3, COPG1, COPZ1, CXCL5, ERN1, EYA4, GSTA1, HLA-J, HYOU1, IFNB1, LCAT, MGLL, NPEPPS, Pdx1, PEX6, PHLDB2, PPARG, PTGES2, RGS14, RNASE1, TBXAS1, TMEM173, TNF, TPM2, Trim30a/Trim30d, XBP1	15	14

^a^The network was based on SWCNT vs. control comparison, *p* < 0.05 and fold changes >2.

^b^Capitalized names indicate genes while lower case names indicate complexes.

^c^The number of differentially expressed genes in each network are reported.

### Aberrant p53 signaling

Our initial study reported B-SWCNT malignant transformed cells possessing an apoptotic resistant phenotype due in part to aberrant p53 signaling [13]. Apoptosis plays an essential role in the removal of mutated or transformed cells and its disruption contributes to abnormal cell growth and malignancy [48,49]. To expand on this finding, we first examined p53 (*TP53*) and p53-associated protein mRNA expression in B-SWCNT compared to B-Control cells. Moderate decreased expression of *TP53* mRNA was observed, but no statistical significance was found (Table 4). Although 13 other p53-associated transcripts showed no statistical difference, three transcripts experienced increased or decreased expression (>twofold) which included *TP53I3*, *TP53INP1*, and *TP53INP2*. This data suggested a moderate alteration of *TP53*-related signaling potentially due to post-transcriptional modifications. Overexpressed *TP53I3*, an oxidoreductase, is typically observed as a response to reactive oxidative species (ROS) stress which has been shown to occur in B-SWCNT cells [10,50]. More interestingly, decreased *TP53INP1* and *TP53INP2* expression suggests a decreased ability in B-SWCNT cells to respond to cell stress. Both genes encode for antioxidant proteins that respond to cell stress by positively regulating autophagy and directly phosphorylating serine 46 on p53 protein which is known to increase p53's apoptotic signaling ability [33]. BEAS-2B cells exhibit increased autophagy in response to ROS stress, including CNT and other inhalable xenobiotic exposure, and dysregulation of autophagy may contribute to tumor promotion potential [50,51]. Decreased expression of these two transcripts suggests that B-SWCNT cells lost two major cell protection mechanisms including a major p53 pro-apoptotic signaling pathway and positive regulation of autophagy.

**Table 4:** **mRNA expressions of** ***TP53*** **and related genes in SWCNT-transformed BEAS-2B cells**

**Probe ID**	**Gene**	**Description**	***p*** **value**	**Fold**
A_23_P26810	TP53	Homo sapiens tumor protein p53 (Li-Fraumeni syndrome)	0.66	−1.48
A_24_P274842	TP53AP1	Homo sapiens mRNA for P53TG1-B, complete cds.	0.75	1.26
A_23_P145895	TP53AP1	Homo sapiens mRNA for P53TG1-C, complete cds.	0.94	1.07
A_23_P88703	TP53BP1	Homo sapiens tumor protein p53 binding protein, 1	0.70	−1.21
A_23_P12526	TP53BP2	Homo sapiens tumor protein p53 binding protein, 2, transcript variant 2	0.17	−1.50
A_23_P150281	TP53I11	Homo sapiens tumor protein p53 inducible protein 11	0.82	−1.75
A_24_P185207	TP53I13	Homo sapiens tumor protein p53 inducible protein 13	0.55	1.61
A_24_P185205	TP53I13	Homo sapiens tumor protein p53 inducible protein 13	0.68	1.44
A_23_P5392	TP53I3	Homo sapiens tumor protein p53 inducible protein 3, transcript variant 1	0.18	2.55
A_23_P168882	TP53INP1	Homo sapiens tumor protein p53 inducible nuclear protein 1	0.10	−4.86
A_24_P357465	TP53INP2	Homo sapiens tumor protein p53 inducible nuclear protein 2	0.53	−2.66
A_24_P245646	TP53RK	Homo sapiens TP53 regulating kinase	0.60	−1.33
A_24_P227971	TP53TG3	Homo sapiens TP53TG3 protein	0.65	1.50
A_23_P49391	TP53TG3	Homo sapiens TP53TG3 protein	0.95	1.07

**Figure 2:**
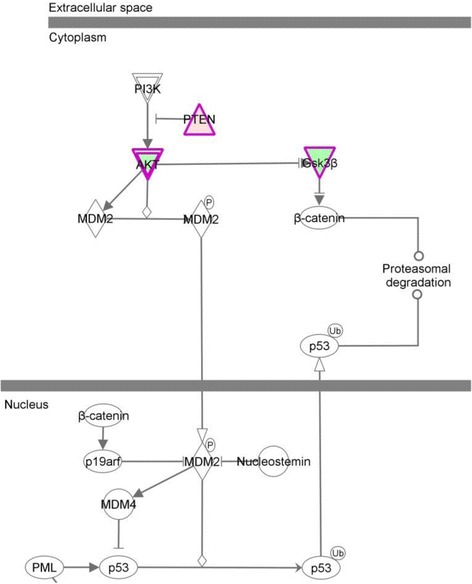
***Akt2*** **,** ***PTEN*** **, and** ***GSK3B*** **-mediated alteration of p53 and β-catenin function in SWCNT transformed BEAS-2B cells.** Color indicates upregulation (in red) and downregulation (in green). Protein assay (Figure 4) showed no significant level changes of Akt proteins and increased phosphorylated Akt indicating that activated Akt may play a role in Gsk3β inhibition of B-catenin and MDM2-mediated p53.

To identify pathways that potentially altered p53 signaling and fate, several canonical pathways with DEGs with known p53 functions were plotted in IPA. Upregulation of *PTEN* and lowered expression of *AKT2* suggested decreased Akt signaling upstream of MDM2 regulation of p53 (Figure 2). Also, decreased *GSK3β* expression suggests release of β-catenin from APC complex resulting in activation of Wnt signaling [52] and a potential block to MDM2-mediated p53 degradation via the proteosome.

**Figure 3:**
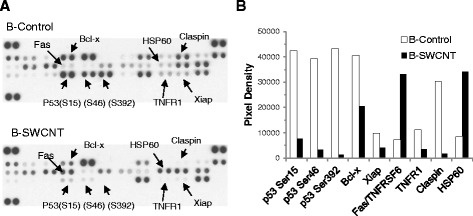
**Apoptosis protein expression array comparison of B-SWCNT to B-Control cells.** Apoptosis protein expression array comparison of B-SWCNT to B-Control cells showed **(A)** altered Bcl-x, Xiap, Fas, TNFR1, claspin, and HSP60 expression in addition to three serine phosphorylation sites on p53 shown in our previous report [13]. **(B)** Densitometry quantification of protein array. Representative data are shown from two independent replicate experiments. See Additional file 1: Table S1 for array layout.

To substantiate post-translational phosphorylation of p53 and identify other proteins contributing to B-SWCNT cell apoptotic resistance ability, we conducted an apoptosis protein expression array. Figure 3 shows, in addition to substantial decreased phosphorylation of Ser46, a target of *TP53INP1*/*2*, Ser15 and Ser392, targets of MDM2 and casein kinase II [13,33,53], which indicated an unstable and dysfunctional p53 response in contributing to B-SWCNT malignant phenotype and enhanced cancer signaling (Table 3, network 7). Other apoptotic-associated proteins were found to alter expression. A substantial decrease in claspin expression indicated loss of checkpoint-mediated ATR control of the cell cycle following DNA damage. Overexpressed heat shock protein 60 (*HSPD1*) in B-SWCNT cells may contribute to cell proliferation and apoptotic resistance in response to genotoxic stress (Table 3, networks 1 and 9) by stabilizing, surviving, and inhibiting p53 [54]. Moderate decreased TNFR1, the major receptor for TNFα, indicated a reduced ability to respond to pro-death TNFα signal which was previously confirmed [13]. Conversely, a fivefold overexpressed Fas receptor in the extrinsic apoptotic pathway suggested a sensitization to apoptosis; however, it is possible that downstream modulators of Caspase 8 activity (i.e., cFLIP) may block this effect. Bcl-xl and Xiap, anti-apoptotic Bcl-2 family proteins, experienced a moderate decrease (twofold) in B-SWCNT compared to B-Control cells. Cells undergoing tumorigenic transformation typically exhibit changes in both pro-survival and death proteins [49]. Investigation of both extrinsic and intrinsic signaling pathways and their role in B-SWCNT apoptotic resistance is currently under investigation in our group.

A recent demonstration of MWCNT-induced mesothelioma in p53 knockout mice [19,55] further supports our finding and the role of p53 in CNT carcinogenesis. A portion of this response may be due to a weakened p53 in the BEAS-2B cell model since the SV40 large T antigen immortalization procedure does affect p53 function [44]. Numerous studies acknowledge that p53 status is a contributing factor to adverse effects following many different types of inhalable and respirable particle exposure to the lung [34,36,38,39,43]. In addition, other studies note that a CNT's physicochemical properties and toxicokinetics in combination with p53 status of exposed tissue determine disease response [56,57]. Regardless, SWCNT exposure did alter p53 signaling in these cells compared to B-Control cells. These findings add to the growing literature that pre-existing p53 condition along with physicochemical properties of CNTs contribute to disease outcome following respirable fiber exposure [19].

**Figure 4:**
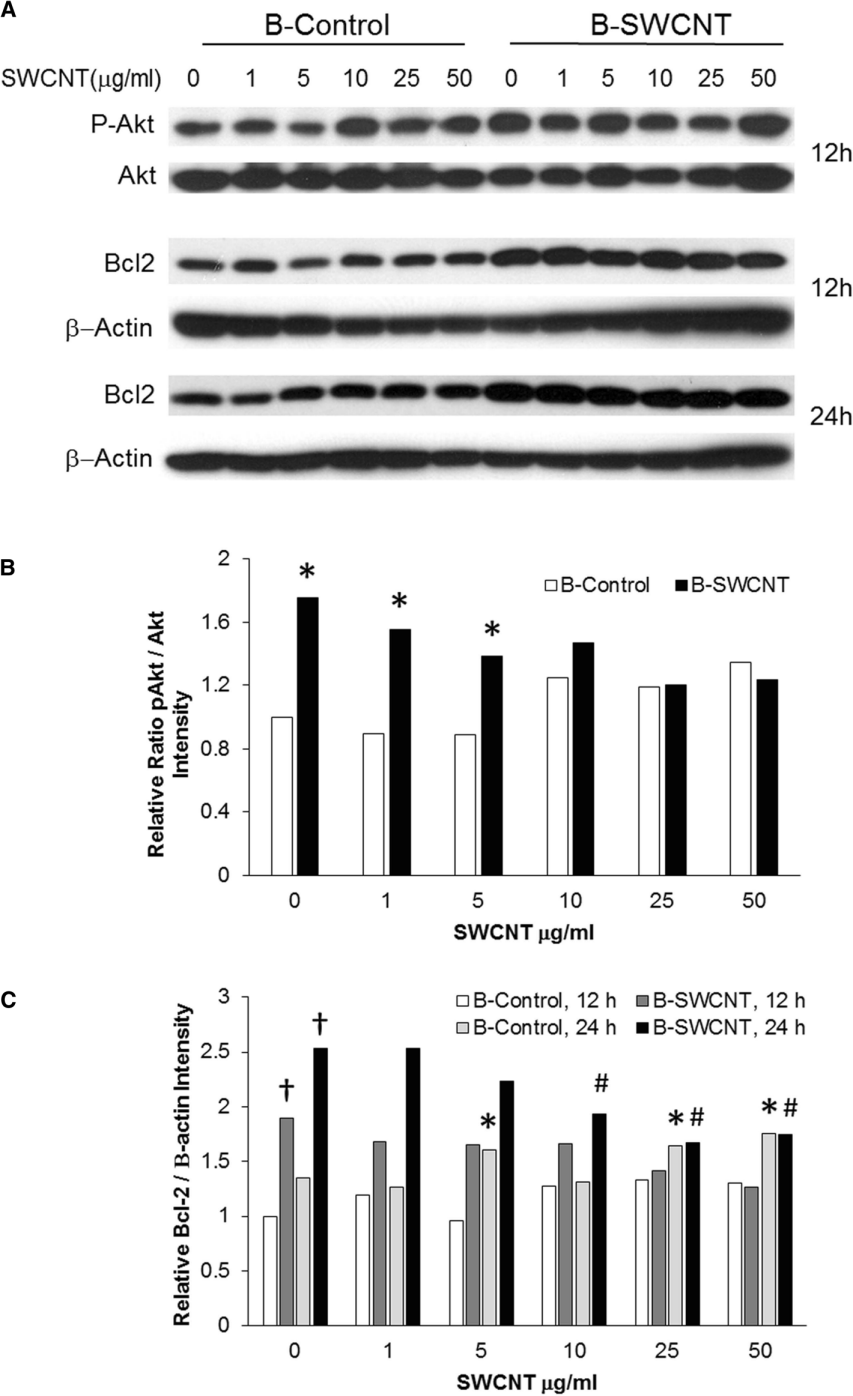
**pAkt, Akt, and Bcl-2 expression via SDS PAGE in B-Control and B-SWNCT transformed cells.** pAkt, Akt, and Bcl-2 expression via SDS PAGE in B-Control and B-SWNCT transformed cells following 12 and 24 h re-exposure to dispersed SWCNT (1 to 50 μg/ml = 0.1 to 5.2 μg/cm2). β-actin was used as an internal loading control. **(A)** Representative blots are shown from three independent experiments. **(B)** pAkt/Akt ratio densitometry quantification. *indicates significant difference between the two cell types (*p* < 0.05). **(C)** Bcl-2 expression densitometry quantification. † indicates significant difference between B-SWCNT and B-Control at each time point (*p* < 0.05). *and # indicate significant difference of both B-Control and B-SWCNT cells, respectively, compared to its unexposed treatment group at 24 h (*p* < 0.05).

Since the above evidence suggested MDM2 activity and *AKT1* is a major signaling protein in most cells compared to *AKT2*, we investigated whether B-SWCNT cells compared to B-Control cells exhibited increased AKT activation via phosphorylation, both in the presence and absence of SWCNT. Western blot analysis indicated increased ratio (1.7-fold, *p* < 0.05) of phosphorylated Akt (pAkt) to Akt protein expression in 0 to 5 μg/ml SWCNT in B-SWCNT compared to B-Control cells (Figure 4A,B). Exposure to SWCNT caused a moderate 20% increase in pAkt levels in B-Control cells at ≥10 μg/ml after 12 h exposure while re-exposure to B-SWCNT exhibited a 27% decrease at high dose levels (≥25 μg/ml). These results show that unexposed lung epithelial cells experience enhanced Akt activation following SWCNT exposure [17]. In addition, it suggested that prolonged, low-dose SWCNT exposure results in a dysregulated, constitutively active pAKT and that Akt signaling may not be regulated at the mRNA level. Akt activation, in turn, potentially activated MDM2, leading to enhanced p53 ubiquitination and potential blockage of GSK3β (Figure 2). Thus, PI3K-AKT signaling may be involved in SWCNT oncogenesis by promoting cell survival (Table 3, network 3). Since pAkt is known to suppress intrinsic mitochondrial pathway and p53 blocks Bcl-2 expression, protein analysis showed that Bcl-2 protein expression was significantly elevated 2.5-fold in B-SWCNT compared to B-Control cells at 0 μg/ml (*p* < 0.05; Figure 4A,C). At 12 h post-exposure to SWCNT, neither cell type displayed a significant change in Bcl-2 expression. Following 24-h SWCNT exposure, B-Control cells displayed a significant increase in Bcl-2 expression at ≥5 μg/ml. Conversely, B-SWCNT displayed a significant decrease in Bcl-2 expression at ≥10 μg/ml. These dose-dependent Bcl-2 expression changes correlated with pAkt levels in each cell type. This suggested that SWCNT exposure to lung epithelial cells first results in Akt activation at 12 h followed by increased Bcl-2 at 24 h, which potentially occurs via either diminished p53 signaling to Noxa [33] or blockage of BAD activity [48]. Persistent stimulation of this pathway could result in an apoptosis resistance phenotype via Bcl-2 overexpression. Interestingly, the dose-dependent drop in pAkt expression mirrored the drop in Bcl-2 expression (−28%) in re-exposed B-SWCNT cells, which resulted in equivalent pAKt and Bcl-2 expression levels in both cell types at high SWCNT doses (≥10 μg/ml). This suggests that pAkt expression sufficiently regulates Bcl-2-associated anti-apoptotic signaling in B-SWCNT cells [48] at low doses that typically occur in occupational exposures. Under unrealistic high doses, however, B-SWCNT cells may partially succumb to pro-apoptotic intrinsic signaling and may rely on other apoptotic resistance mechanisms (e.g., dysfunctional extrinsic pathway). In summary, prolonged SWCNT exposure coupled with p53 instability resulted in overexpressed pAkt and Bcl-2 which potentially plays a role in the apoptotic resistance and oncogenic development of B-SWCNT cells.

### Genes involved in the molecular mechanism of cancer

Among the various possible molecular mechanisms of cancer, as shown in Additional file 2: Figure S1, both increased (*RASGRP*, *ARHGEF9, SHC*, and *RHOB*) and decreased gene expressions (*RBPJK*, *JNK*, *DVL1*, *GSK3A*, *GSK3β*, *PAK*, *AKT2, PMAIP1* [Noxa], *BAX*) were observed in B-SWCNT cells. Based on overexpressed pAkt, decreased stabilization of p53 via several serine dephosphorylations and increased Bcl-2 protein, it appears that B-SWCNT cells gained apoptotic resistance in the intrinsic apoptotic pathway via the pAkt/MDM2/p53/Bcl-2 signaling axis. With the decreased pro-apoptotic *BAX and PMAIP1 (*Table 3, networks 3 and 9) expression downstream of pAkt, and our elevated levels of Bcl-2 protein (Western blot data), we postulated this anti-apoptotic signaling mechanism in the intrinsic apoptosis pathway of B-SWCNT cells.

Several DEGs in other signaling pathways potentially contributed to B-SWCNT malignant phenotype. Increased expression of *RASGRP1* and RasGEF (*ARHGEF9*) may contribute to Ras-ERK signaling that regulates cyclin expression and thus cell cycle progression (Table 3, network 1). Increased *SHC* and Rho (*RHOB*) expression may also activate Cdc42, a key cell cycle regulator, and contribute to PI3K/Akt activation. It was reported that smoke-concentrated medium exposure induced Cdc42 translocation in human bronchial epithelial cells that may contribute to lung carcinogenesis [58]. Downregulation of Dsh (*DVL1;* Table 3, network 3) may remove its blockage of Notch1, which could trigger notch signaling-mediated tumorigenesis [59] and p53-dependent carcinogenesis [60]. Decreased *GSK3A* and *GSK3β* expression (Table 3, networks 3 and 9) potentially enhanced β-catenin tumor promotion and increased Cyclin D protein levels leading to enhanced G1 phase transition and accelerated cell proliferation. It is believed that GSK-3β regulates Wnt signaling pathway and its aberrant activation often results in tumor formation [52]. In this study, downregulated GSK3β in APC complex (a prominent anti-lung cancer mediator), at the transcript or post-transcriptional level via pAkt activation, may lead to dysfunctional β-catenin and thus potential elevated Wnt signaling (Figure 4). Lastly, downregulation of JNK, a MAPK family kinase, does not seem to support Fos/Jun-mediated transcription in SWCNT carcinogenic potential. However, decreased JNK can result in decreased phosphorylation of p53 Ser15, resulting in MDM2-mediated p53 degradation [61].

## Conclusions

In conclusion, we observed that long-term exposure *in vitro* (6 months) to SWCNT caused malignant transformation of human lung epithelial cells as suggested by our genome-wide expression data. Several genes involved in the apoptosis, cell cycle control, and oncogenic development were altered by the SWCNT treatment with the immune response representing the most altered biological process. The oncogenic phenotype of transformed B-SWCNT cells [13] may be explained, among other possible mechanisms, by active pAkt signaling, destabilized p53, increased expression of Ras family genes, Dsh-mediated Notch1, overexpressed Bcl-2 and downregulation of the anti-apoptotic genes *BAX* and *PMAIP1*. The development of the sub-chronic *in vitro* exposure model coupled with global transcriptome analysis described in this study could aid in the investigation of the potential mechanisms of CNT carcinogenesis, while the genome-wide expression approach can provide new insights into the genes involved in the carcinogenic process [40].

## Additional files

Additional file 1: Table S1. Layout of apoptosis protein expression array (R&D Systems). Additional file 2: Figure S1. Differentially expressed genes in the molecular mechanism of cancer pathway in SWCNT-transformed BEAS-2B cells. Colors indicate under-(green) and over-expressed (in red) genes compared to unexposed passage control cells. Values are fold changes of SWCNT-treated vs. control, *p* ≤ 0.05. The double colors indicate both increased and decreased genes within the same group (e.g., kinases).

## Abbreviations

CNTs: carbon nanotubes
SWCNT: single-walled carbon nanotubes
MW: multi-walled
SW: single-walled
B-SWCNT: transformed BEAS-2B cells following SWCNT exposure
B-Control: passage matched, unexposed BEAS-2B cells
DEGs: differentially expressed genes
GO: gene ontology
IPA: Ingenuity Pathway Analysis
ROS: reactive oxygen species
