# Supplementary material for: Gene expression profile of human lung epithelial cells chronically exposed to single-walled carbon nanotubes
Source: Nanoscale Res Lett. 2015 Jan 27;10:12. doi: 10.1186/s11671-014-0707-0 (PMC4314466; doi:10.1186/s11671-014-0707-0)
Supplement: Additional file 1: Table S1. — Layout of apoptosis protein expression array (R&D Systems). [file 11671_2014_707_MOESM1_ESM.docx]

**Additional file**

**Table S1.** Layout of apoptosis protein expression array (R&D Systems).

| Row | 1,2 | 3,4 | 5,6 | 7,8 | 9,10 | 11,12 | 13,14 | 15,16 | 17,18 | 19, 20 | 21,22 | 23,24 |
| --- | --- | --- | --- | --- | --- | --- | --- | --- | --- | --- | --- | --- |
| A | reference | |  |  |  |  |  |  |  |  |  |  |
| B | Bad | Bax | Bcl2 | Bclx | Pro-Caspase 3 | Cleave Caspase 3 | Catalase | cIAP 1 | cIAP 2 | Claspin | Clusterin | Cytochrome C |
| C | TRAIL DR4 | TRAIL DR5 | FADD | Fas | HIF-1A | HO-1 | HO-2 | HSP27 | HSP60 | HSP70 | HTRA2 | Livin |
| D | PON2 | p21 | p27 | p-p53Ser15 | p-p53Ser46 | p-p53Ser392 | p-Rad17 Ser635 | SMAC/Diablo | Survivin | TNF R1 | XIAP | PBS |
