# Supplementary material for: Gene expression profile of human lung epithelial cells chronically exposed to single-walled carbon nanotubes
Source: Nanoscale Res Lett. 2015 Jan 27;10:12. doi: 10.1186/s11671-014-0707-0 (PMC4314466; doi:10.1186/s11671-014-0707-0)
Supplement: Additional file 2: Figure S1. — Differentially expressed genes in the molecular mechanism of cancer pathway in SWCNT-transformed BEAS-2B cells. Colors indicate under-(green) and over-expressed (in red) genes compared to unexposed passage control cells. Values are fold changes of SWCNT-treated vs. control, p ≤ 0.05. The double colors indicate both increased and decreased genes within the same group (e.g., kinases). [file 11671_2014_707_MOESM2_ESM.pdf]

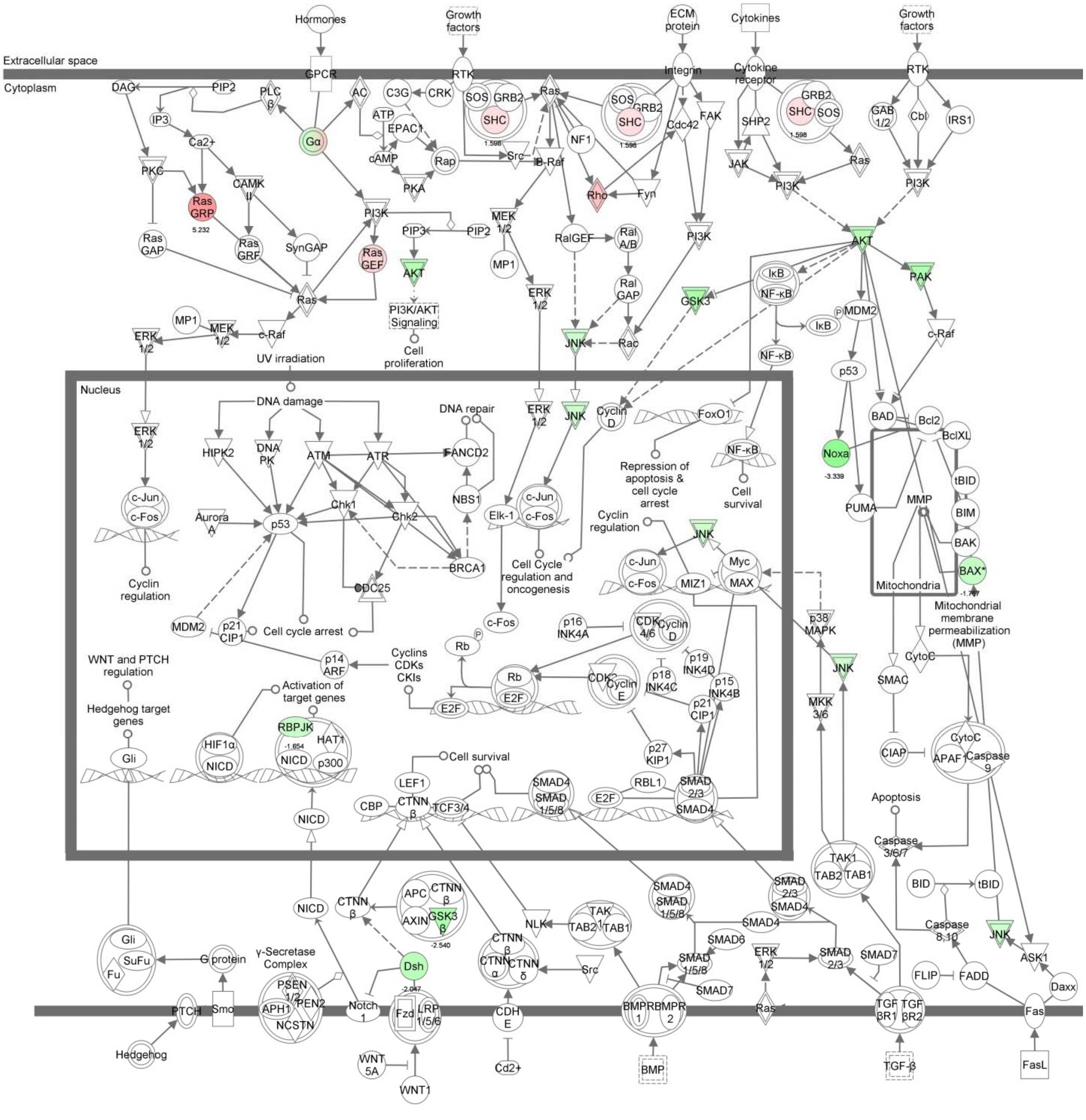
Hormones
Growth factors
ECM protein
Cytokines
Growth factors
Extracellular space
Cytoplasm
GPCR
RTK
Integrin
Cytokine receptor
RTK
DAG
PIP2
PLC β
AC
C3G
CRK
SOS
GRB2
SHC
1.598
Ras
SOS
GRB2
SHC
1.598
GRB2
SHC
SOS
1.598
GAB 1/2
Cbl
IRS1
IP3
Ca2+
Gα
ATP
EPAC1
cAMP
Rap
Src
B-Raf
NF1
Cdc42
FAK
SHP2
JAK
PI3K
Ras
PI3K
PKC
Ras GRP
5.232
CAMK II
PKA
Rho
Fyn
PI3K
MEK 1/2
Ras GAP
Ras GRF
SynGAP
Ras GEF
PIP3
PIP2
AKT
MP1
RalGEF
Ral A/B
PI3K
AKT
PAK
Ras
PI3K/AKT Signaling
Cell proliferation
ERK 1/2
Ral GAP
GSK3
IκB
NF-κB
IκB
MDM2
c-Raf
MP1
MEK 1/2
c-Raf
ERK 1/2
JNK
Rac
NF-κB
UV irradiation
p53
Nucleus
DNA damage
DNA repair
ERK 1/2
JNK
Cyclin D
FoxO1
BAD
Bcl2
BclXL
ERK 1/2
HIPK2
DNA PK
ATM
ATR
FANCD2
Repression of apoptosis & cell cycle arrest
NF-κB
Noxa
-3.339
tBID
c-Jun
c-Fos
NBS1
Chk1
c-Jun
c-Fos
Elk-1
Cell survival
PUMA
MMP
BIM
Aurora A
p53
Chk2
Cyclin regulation
BAK
JNK
BRCA1
Cell Cycle regulation and oncogenesis
c-Jun
c-Fos
MIZ1
Myc
MAX
BAX*
-1.747
Mitochondria
Cyclin regulation
CDC25
Mitochondrial membrane permeabilization (MMP)
MDM2
p21 CIP1
Cell cycle arrest
c-Fos
p16 INK4A
CDK 4/6
Cyclin D
p38 MAPK
Rb
P
CytoC
WNT and PTCH regulation
p14 ARF
Cyclins CDKs CKIs
p19 INK4D
JNK
SMAC
Rb
E2F
CDK2
Cyclin E
p18 INK4C
p15 INK4B
Hedgehog target genes
Activation of target genes
E2F
p21 CIP1
MKK 3/6
RBPJK
-1.654
HAT1
HIF1α
NICD
NICD
p300
p27 KIP1
CIAP
CytoC
APAF1
Caspase 9
Gli
Cell survival
LEF1
SMAD4
SMAD 1/5/8
RBL1
SMAD 2/3
SMAD4
Apoptosis
CBP
CTNN β
TCF3/4
E2F
NICD
Caspase 3/6/7
TAK1
TAB2
TAB1
SMAD4
SMAD 1/5/8
SMAD 2/3
SMAD4
BID
tBID
APC
CTNN β
NICD
CTNN β
AXIN
GSK3 β
-2.540
TAK 1
TAB2
TAB1
NLK
SMAD4
Caspase 8,10
JNK
Gli
SuFu
Fu
γ-Secretase Complex
CTNN β
CTNN α
CTNN δ
SMAD6
SMAD 1/5/8
ERK 1/2
SMAD 2/3
SMAD7
G protein
Dsh
-2.047
Src
FLIP
FADD
ASK1
Daxx
PSEN 1/2
APH1
PEN2
NCSTN
SMAD7
PTCH
Smo
Notch 1
Fzd
LRP 1/5/6
CDH E
BMPR 1
BMPR 2
Ras
TGF βR1
TGF βR2
Fas
Hedgehog
WNT 5A
WNT1
Cd2+
BMP
TGF-β
FasL

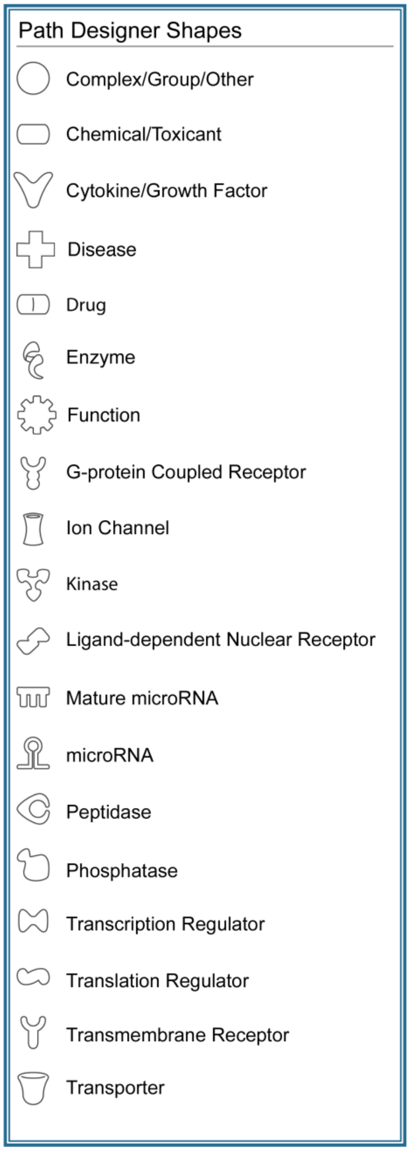

Supplement
Figure legend for the Figure 1s (IPA)
